# Updates on the Physiopathology of Group I Metabotropic Glutamate Receptors (mGluRI)-Dependent Long-Term Depression

**DOI:** 10.3390/cells12121588

**Published:** 2023-06-08

**Authors:** Dalila Mango, Ada Ledonne

**Affiliations:** 1 School of Pharmacy, University of Rome “Tor Vergata”, 00133 Rome, Italy; dalilamango@gmail.com; 2 Laboratory of Pharmacology of Synaptic Plasticity, European Brain Research Institute, 00161 Rome, Italy; 3 Department of Systems Medicine, University of Rome “Tor Vergata”, 00133 Rome, Italy; 4 Department of Experimental Neuroscience, IRCCS Fondazione Santa Lucia, 00143 Rome, Italy

**Keywords:** mGluR1, mGluR5, synaptic plasticity, glutamatergic transmission, AMPARs, NMDARs, long-term depression, learning and memory, neuropsychiatric diseases, neurodevelopmental disorders

## Abstract

Group I metabotropic glutamate receptors (mGluRI), including mGluR1 and mGluR5 subtypes, modulate essential brain functions by affecting neuronal excitability, intracellular calcium dynamics, protein synthesis, dendritic spine formation, and synaptic transmission and plasticity. Nowadays, it is well appreciated that the mGluRI-dependent long-term depression (LTD) of glutamatergic synaptic transmission (mGluRI-LTD) is a key mechanism by which mGluRI shapes connectivity in various cerebral circuitries, directing complex brain functions and behaviors, and that it is deranged in several neurological and psychiatric illnesses, including neurodevelopmental disorders, neurodegenerative diseases, and psychopathologies. Here, we will provide an updated overview of the physiopathology of mGluRI-LTD, by describing mechanisms of induction and regulation by endogenous mGluRI interactors, as well as functional physiological implications and pathological deviations.

## 1. Group 1 Metabotropic Glutamate Receptors (mGluRI)

mGluR1 and mGluR5 are G protein-coupled receptors (GPCR) constituting the group I metabotropic glutamate receptor (mGluRI). mGluR1 is present in four isoforms (mGluR1α, β, γ, δ) whereas mGluR5 exists in two variants (mGluR5α and mGluR5β). The various isoforms, arising by alternative splicing, mainly differ in the C-terminal intracellular tail [1]. The mGluRI distinction from other mGluR groups (group II, including mGluR2 and mGluR3, and group III, comprising mGluR4, mGluR6, mGluR7, mGluR8) is based on amino-acid homology, agonist binding, and signaling pathways downstream to receptor activation [1].

**Figure 1:**
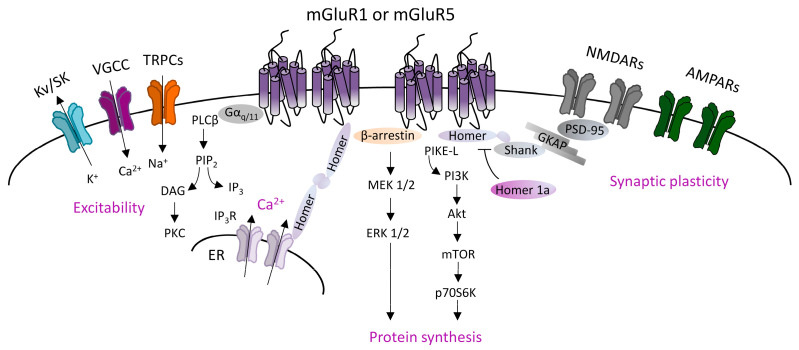
Group 1 metabotropic glutamate receptors (mGluRI) signaling. Scheme of the principal mGluR1 and mGluR5 signaling pathways, showing that G_q/11_-dependent activation of phospholipase C β (PLCβ) mediates phosphatidylinositol hydrolysis with the generation of diacylglycerol (DAG) (that activates protein kinase C, PKC) and inositol-1,4,5-trisphosphate (IP_3_) (that fosters Ca^2+^ intracellular release from internal stores by acting on IP_3_R receptors on the endoplasmic reticulum). mGluR1/5, through G_q/11_-dependent mechanisms, also modulates ion channels, such as transient receptor potential channels (TRPCs), voltage-gated Ca^2+^ channels (VGCC), and different types of K^+^ channels (K_v_ or SK), thus affecting neuronal excitability. Additional G protein-independent mechanisms involve the recruitment of β-arrestin or other scaffolding/adaptor proteins such as Homer long isoforms, that provide mGluRI coupling with other effectors, thus fostering activation of signaling pathways, such as the phosphatidylinositol 3-kinase/Akt/mammalian target of rapamycin (PI3K-Akt-mTOR) kinase pathway, or MEK1/2-ERK 1/2 pathway, both involved in mechanisms promoting protein synthesis.

Canonical mGluRI signaling is driven by the G_q_/G_11_-dependent activation of phospholipase C β (PLCβ), inducing the hydrolysis of phosphoinositides and generation of inositol 1,4,5-trisphosphate (IP_3_) and diacyl-glycerol (DAG). This pathway leads to the Ca^2+^ intracellular mobilization from internal stores and activation of protein kinase C (PKC) [1]. In addition, mGluRI can activate several other pathways, and also G protein-independent mechanisms, by means of the interaction with specific adaptor proteins that recruit distinct signaling components [1]. G protein-independent mechanisms mainly lie on β-arrestin binding, favored by the receptor phosphorylation by G-protein-coupled receptor kinases (GRKs). Globally, mGluRI can activate a multifaceted list of effectors, including phospholipase D (PLD), protein kinases pathways such as mitogen-activated protein kinase/extracellular receptor kinase (MAPK/ERK), the mammalian target of rapamycin (mTOR)/p70S6 kinase pathway, casein kinase 1, cyclin-dependent protein kinase 5, and Jun kinase (JUNK) [1,2] (Figure 1).

mGluR1 and mGluR5 are mainly localized in the postsynaptic densities (PSD) in perisynaptic zones [3,4,5], where they form multiprotein complexes by interacting with other membrane proteins and downstream effectors. Different intracellular scaffolding proteins interacting with mGluR1 and mGluR5 have been identified. An important group is constituted by the family of Homer proteins, including long isoforms (Homer 1b, 1c, 2, 3), able to make a large multimeric assembly in the PSD, and the shorter isoform Homer 1a, that does not form protein complexes but antagonizes Homer longer isoforms connections [6,7]. Homer longer isoforms bond mGluR1 or mGluR5 to their principal signaling effectors, such as PLC, PI3K and the PI3K enhancer, PIKE-L, as well as the IP_3_ receptor, and various classes of ion channels including transient receptor potential-like channels (TRPCs), voltage-gated calcium channels (VGCC), and M-type potassium channels [8,9,10]. Moreover, Homer long isoforms, by linking other scaffolding proteins (most notable are PSD-95 and Shank), directly associate mGluR1/5 to other membrane receptors, including NMDARs. Besides the Homer family, other identified mGluR1- and mGluR5-interacting proteins include tamalin, norbin, preso-1, calmodulin, neuronal calcium-binding protein 2 (NECAB2), calcineurin inhibitor protein (CAIN), the ubiquitin ligase Siah-1A, various protein kinases, including PKC, GRK2, CaMKII, and cytoskeletal components, such as 4.1 G and Filamin-A [8,9,10].

mGluR1 and mGluR5 also bind intracellular regulatory proteins, such as GPCR kinases (GRKs) (GRK2 and GRK3 subtypes for mGluR5, and GRK2, GRK4, and GRK5 for mGluR1) [11,12,13], which control mGluRI internalization by phosphorylation [14], or members of the family of the regulator of G-protein signaling (RGS) (such as RGS-4), which increase the GTPase activity of Gα_q_ and uncouple G protein-linked effectors, and thus switch-off mGluRI signaling [15].

Based on common signaling mechanisms, mGluR1 and mGluR5 have been often regarded as interchangeable, but it is now overt that they can either have separate functions [16,17] or be cross-talking, by acting occasionally in a cooperative [18] or otherwise antagonistic manner [19,20]. mGluR1 and mGluR5 are active as homodimers, i.e., mGluR1-mGluR1 and mGluR5-mGluR5, and additionally the two subtypes can be associated among them in the heterodimer mGluR1-mGluR5, or with other GPCRs belonging to adenosine-, GABA-, and dopamine receptors [21,22,23,24,25,26], by forming dimers such as mGluR1-A1 [23], mGluR1-GABA_B_ [24], and mGluR5-D1 [25], or a trimeric receptor complex, such as mGluR5-A2A-D2 [26]. Furthermore, mGluR1/5 can assembly with ligand-gated ion channels, such as NMDARs [27], and interplay with other ion channels beyond TRPCs, N-type Ca^2+^ channels, and M-type K^+^ channels, such as small conductance calcium-activated potassium (SK) channels [28,29], and acid-sensing ion channels 1a (ASIC1a) [30]. Furthermore, mGluR1/5 cross-talk with receptor tyrosine kinases (RTKs), such as epidermal growth factor receptor (EGFR) and ErbBs, that are receptors for the neurotrophic factors EGF and neuregulins, respectively [31].

mGluR1 and mGluR5 are often co-expressed in the various brain areas and cellular populations, but they can also have different levels or segregate expression in distinct cellular populations [32,33]. Though mGluR1 and mGluR5 are usually described as postsynaptic, they can also be presynaptic [33], and are expressed in astrocytes and microglial cells in several brain areas in physiological and pathological conditions [34], thus implying a more complex scenario of potential mGluR1/5-engaged mechanisms. Furthermore, in addition to plasma membrane-anchored receptors, functional mGluR1/5 pools are located in intracellular compartments, in the endoplasmic reticulum (ER), nuclear, and mitochondrial membranes, wherein they can activate different signaling pathways, thus supporting the cytoplasm-to-nuclear translocation or mGluR1/5 mitochondrial functions [35,36,37].

In conclusion, mGluR1- and mGluR5 interplay with other membrane receptors, ion channels, intracellular scaffolding proteins, regulatory proteins, and signaling pathways, as well as differences in their cellular and sub-cellular localization rule definite roles in different contexts, shaping mGluR1/5 impact on brain functions.

## 2. mGluRI-Dependent LTD in Physiology

mGluR1 and mGluR5 manage important brain functions, by affecting neuronal excitability, intracellular Ca^2+^ dynamics, protein synthesis, dendritic spines maturation, and synaptic transmission. They are prime actors in the induction of synaptic plasticity, mediating either long-term potentiation (LTP) or long-term depression (LTD) of synaptic transmission driven by diverse neurotransmitters systems, thus fine-tuning glutamatergic, GABAergic, and dopaminergic transmission.

mGluRI-dependent LTD of glutamatergic synaptic transmission (mGluRI-LTD)—a widely studied form of synaptic plasticity—underlies essential brain functions, such as learning and memory processes and complex behaviors. In this section, we first describe mGluRI-LTD’s mechanisms of induction and regulation, and then report insights on mGluRI-LTD physiological implications.

### 2.1. Mechanisms Underlying mGluRI-LTD

mGluRI-LTD is inducible in ex vivo brain slices preparations or in vivo rodents by pharmacological mGluRI activation with the mGluR1/5 agonist 3,5-dihydroxyphenylglycine (DHPG) (chemical mGluRI-LTD), by electrical stimulations of synaptic terminals promoting glutamate release and mGluRI activation (synaptic mGluRI-LTD) [18,38,39,40,41], or as recently reported by optogenetic activation of mGluR1 signaling [42]. mGluRI-LTD has been described in various brain areas, including cerebellum, hippocampus, dorsal striatum, nucleus accumbens, ventral tegmental area, substantia nigra pars compacta, lateral habenula, and cortex. Suitable conditions for mGluRI-LTD induction in different brain areas/synapses are reliant on adjustments of DHPG concentration/treatment’s duration, or stimulating protocols, typically represented by electrical single- or paired-pulse (PP) prolonged low-frequency stimulation (LFS) at 1 Hz for 10–15 min, or occasionally shorter high-frequency stimulation (HFS) protocols [18,38,39,40,41,43,44].

**Figure 2:**
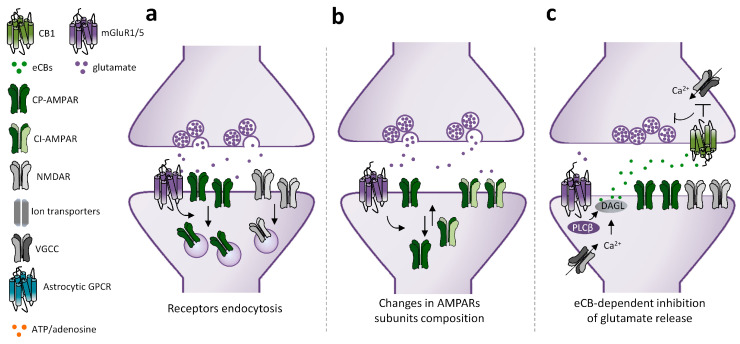

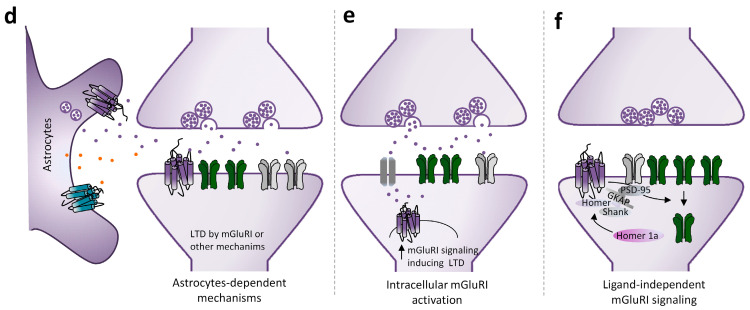
Mechanisms underlying mGluRI-LTD. Scheme of mGluRI-dependent mechanisms underlying glutamatergic LTD. (**a**) mGluRI activation induces AMPARs or NMDARs internalization, thus decreasing glutamatergic synaptic currents; (**b**) mGluRI activation promotes a switch of membrane-exposed AMPARs, by inducing retrieval of Ca^2+^ permeable AMPARs (CP-AMPARs) and exposition of Ca^2+^ impermeable AMPARs (CI-AMPARs), that have a minor ion conductivity; (**c**) mGluRI stimulation triggers endocannabinoids (eCBs) synthesis in the postsynaptic compartment. Then eCBs, by acting on presynaptic CB1 receptors, negatively control glutamate release; (**d**) activation of mGluRI or other GPCR expressed on astrocytes promote release of gliotransmitters, such as glutamate and ATP, that then induce LTD by acting on postsynaptic mGluRI or adenosine receptors; (**e**) activation of intracellular mGluRI, by glutamate entering in the cytoplasm by membrane transporters, induces classical signaling pathways underlying mGluRI-LTD; (**f**) mGluRI constitutive activity, independent of ligand-induced activation, stimulates signaling pathways underlying LTD.

While the final outcome of mGluRI activation converges in downscaling of glutamatergic transmission, cellular and molecular mechanisms underlying mGluRI-LTD are multiple and composite. Conventional mechanisms underlying mGluRI-LTD are triggered by three main processes: (1) mGluRI-induced endocytosis of ionotropic glutamate receptors (iGluRs), AMPARs, or NMDARs; (2) mGluRI-dependent modifications of AMPARs’ subunits composition; and (3) mGluRI-driven endocannabinoids (eCBs)-dependent reduction of glutamate release (Figure 2). Internalization of AMPARs is a common mechanism by which mGluRI activation produces glutamatergic LTD. AMPARs endocytosis lies behind mGluRI-LTD at parallel fibers-purkinje cells (PF-PC) synapses in the cerebellum and at CA3-CA1 synapses of the hippocampus, despite being achieved by the activation of distinct mGluRI signaling pathways [30]. In cerebellar PCs, AMPARs endocytosis is triggered by a PKC-dependent GluR2 phosphorylation, which decreases subunit affinity for the scaffolding protein GRIP, thus weakening AMPAR membrane docking and favoring its endocytosis [45,46]. Differently, AMPARs endocytosis underlying hippocampal mGluRI-LTD requires the activation of protein kinases other than PKC, such as ERK1/2, PI3K-Akt-mTOR, and MAPKs [47,48,49], that promote synthesis of proteins leading to AMPARs membrane retrieval. NMDARs endocytosis underlies mGluRI-LTD of NMDARs-mediated transmission at hippocampal CA3-CA1 synapses [50,51]. It occurs through an mGluR1/5-activated mechanism not requiring tyrosine kinases or phosphatases, neither intracellular Ca^2+^ mobilization or protein synthesis, but possibly due to a NMDAR lateralization in PSD [51].

Beyond iGluRs endocytosis—in which the net number of functional receptors on surface membrane is reduced—mGluRI can weaken glutamatergic synaptic transmission by inducing a “switch” of AMPARs, with the surface exposition of receptors having a different subunit composition and minor ion permeability with respect to native ones that are retrieved. Such mechanism underlies mGluRI-LTD in ventral tegmental area (VTA) dopamine neurons [52] and in nucleus accumbens (NAc) medium spiny neurons (MSNs) [53], wherein mGluR1 stimulation fosters removal of native GluA1-containing AMPARs (Ca^2+^ and Na^+^ permeable), and insertion of AMPARs having GluA2 subunits, conferring Ca^2+^ impermeability and lower receptor conductibility. 

A different mechanism by which mGluRI depresses glutamatergic transmission is through the regulation of glutamate release. This is commonly achieved by mGluRI-induced eCB synthesis in the postsynaptic compartment, and consequent activation of presynaptic CB1 receptors. The eCB-induced glutamate reduction underlies mGluRI-LTD in the dorsal striatum (dST) [54] and partakes in mGluRI-LTD induction in cerebellar [55] and cortical areas [56].

In addition to such conventional mechanisms, mGluRI-induced LTD could also be reliant on further mechanisms related to mGluRI interplay with other GPCR or receptors/ion channels, or receptors locations in subcellular compartments. Of note, besides the surface extracellular membrane, a large pool of functional mGluR5 is associated with intracellular membranes [35,36,37,57,58]. In the striatum, the activation of such intracellular pool, by glutamate entering in neurons via Na^+^ or Cl^−^-dependent transporters/exchangers, triggers a cascade of molecular events starting with the phosphorylation of ERK1/2 and Elk-1 [36], followed by increased expression of synaptic plasticity genes c-fos, egr-1, and Arc [59]. Thus, intracellular mGluR5 activation could trigger the cascade of events underlying synaptic plasticity. Intracellular mGluR5 have also been reported in hippocampal CA1 pyramidal neurons, associated with intracellular membranes of the ER and nucleus, where they colocalize with the Na^+^-dependent excitatory amino acid transporter EAAT3 [60]. It has been proposed that intracellular mGluR5 activation in hippocampal CA1 pyramidal neurons contributes to LFS-induced LTD induction [60]. Knowledge of the physiological conditions fostering intracellular mGluR1/5 activation is still limited, but it can be speculated that intracellular mGluR1/5 activation could be enabled in conditions that boost glutamate release to levels exceeding membrane glutamate receptors binding and/or when ion/amino acid transporters (mediating glutamate influx also in the postsynaptic compartment) are highly expressed. Regarding mGluRI-LTD induction, intracellular mGluR1/5 activation could be fostered by stimulation protocols allowing a glutamate spillover (such as prolonged LFS or brief HFS), rather than during chemical LTD, due to DHPG’s limited membrane diffusion.

While in the above described mGluRI-LTD mechanism the ligand-driven mGluRI activation represents the first event triggering downstream plastic modifications, some evidence suggests that mGluRI might induce glutamatergic synaptic depression also by other unconventional mechanisms. Actually, a ligand-independent form of mGluRI-dependent synaptic downscaling has been described, in which mGluRI activation is not due to glutamate binding, but rather by the induction of the IEG Homer1a in the postsynaptic neuron, which autonomously fosters mGluRI signaling and AMPARs endocytosis [61,62].

Remarkably, while mGluRI-LTD is typically considered an exclusive neuronal process, it should be considered that mGluR1/5 is also expressed on glial cells—astrocytes and microglia—in different brain areas, thus inferring that glial mGluR1/5-dependent mechanisms could contribute to shaping mGluRI-LTD expression. Consistent evidence points to astrocytes’ essential roles in the active regulation of different forms of synaptic transmission and plasticity by gliotransmitters release [63,64]. Regarding specific astrocytes roles in mGluRI-dependent LTD, it has been recently reported that astrocyte mGluR5 signaling, by inducing ATP release, shapes HFS-induced LTD in striatal MSNs of direct pathways, consequent to adenosine A1 receptors activation [65]. Additionally, mGluR1-induced ATP release from astrocytes modulates mGluRI-LTD magnitude at hippocampal CA3-CA1 synapses and cortical layer 2/3 synapses [66].

In conclusion, the current picture identifies different cellular and molecular mechanisms underlying mGluRI-LTD (Figure 2). They can be entirely confined to the postsynaptic compartment (as in the case of iGluRs endocytosis, AMPARs subunits changes, or intracellular mGluRI activation) or be jointly expressed between post- and presynaptic sides (as for eCB-dependent LTD), as well as involve astrocytes interplay, feasibly via astrocytes-released gliotransmitters. Even though earlier evidence mainly supports brain area- and synapse-specific segregation of mechanisms underlying diverse mGluRI-LTD types, future research may unmask additional overlying, especially embracing the less-investigated mechanisms involving astrocytes, and eventually microglia interplay.

### 2.2. mGluRI Partners Affecting mGluRI-LTD

Theoretically, mGluRI-LTD magnitude is governed by all cellular events that control mGluRI expression, membrane docking, and signaling efficiency. Hence, mGluRI interplay with other GPCR, ligand-gated receptors, ion channels, and receptor tyrosine kinases, as well as intracellular protein–protein interaction, or contact with extracellular-matrix adhesion molecules can temper mGluRI-LTD expression. In spite of the increasing list of mGluRI-interacting proteins, the current knowledge of precise functional impacts of each protein–protein interaction in mGluRI-LTD induction is still partial. Nevertheless, the emergent evidence supports that mGluR1 or mGluR5 partners can have a significant functional effect on proper mGluRI-LTD induction. 

As mentioned, postsynaptic scaffolding proteins can directly connect mGluR1 and mGluR5 with either signaling effectors or other PSD proteins, allowing the formation of macromolecular proteins complex in PSD. Homer long isoforms primarily fulfill this function, by allowing mGluRI bond to several effectors, and localization in the PSD. Homer1a, by the disruption of mGluRI-long Homer cross-linking, causes the constitutive, agonist-independent activity of mGluR1 and mGluR5 [61] and reduces the surface AMPARs expression [62]. Further evidence demonstrates contributions of other adaptor or scaffolding proteins to mGluR1/5-dependent LTD. Tamalin is an mGluR1- and mGluR5-interacting protein subservient receptor intracellular trafficking, membrane expression, and signaling [67,68,69]. Recent evidence documents that tamalin is intrinsically involved in mechanisms underlying hippocampal mGluRI-LTD at CA3-CA1 synapses [70]. Indeed, the intracerebral injection in rats of cell-permeable peptides designed to interfere with the tamalin–mGluRI bond impairs hippocampal mGluRI-LTD expression, mainly opposing mGluR5 function, thus proving that the mGluR5–tamalin interaction is required for this form of hippocampal synaptic plasticity [70].

Norbin is an mGluR5-interacting protein that increases the cell surface localization of the receptor and subsequently mGluR5 signaling [71]. Norbin genetic deletion in mice reduces mGluRI-LTD magnitude at hippocampal CA3-CA1 synapses, indicating that the norbin–mGluR5 bond allows proper hippocampal mGluRI-LTD induction [71].

Calmodulin (CaM) is a major Ca^2+^ sensor protein [72], which also binds and stabilizes mGlu1/5 surface expression [73,74]. CaM activity is required for mGluRI-LTD at hippocampal CA3-CA1 synapses, since CaM pharmacological inhibition impairs DHPG-induced LTD in hippocampal slices and in anesthetized mice [75]. 

CaMKIIα interacts with both mGluR1 and mGluR5, regulating their endocytosis and signaling [76]. While traditionally linked to long-term potentiation, it is emerging that CaMKII activity is also required for glutamatergic LTD forms, including hippocampal mGluRI-LTD. Actually, pharmacological and genetic CaMKII inhibition counteracts mGluRI-LTD induction at hippocampal CA3-CA1 synapses [77,78].

Caveolin-1 (Cav-1) is a protein associated with lipid rafts in caveolae and involved in receptor endocytosis. Cav-1 binds mGluRI in hippocampal pyramidal neurons, influencing receptors constitutive internalization and signaling [79]. Evidence from Cav-1 knockout mice supports that Cav-1 is required for mGluRI-LTD induction at hippocampal CA3-CA1 synapses [80]. 

Spinophilin is a mGluR1- and mGluR5-interacting protein that regulates receptor membrane expression, affecting mGluR1/5 roles in synaptic plasticity. Actually, spinophilin genetic deletion in mice enhances mGluR5 endocytosis and impairs DHPG-induced LTD at hippocampal CA3-CA1 synapses [81]. 

mGluRI interplay with ion channels and ligand-gated receptors can also affect proper mGluRI-LTD expression. TRPCs are a family of non-selective cation membrane channels widely distributed in the brain. TRPC1 subtype contributes to hippocampal mGluRI-LTD modulation, as pharmacological and genetic TRPC1 inhibition reduces hippocampal mGluRI-LTD magnitude [82]. Furthermore, TRPC inhibition also affects the extinction of spatial learning [82], proposed to be correlated with mGluRI-LTD impairment.

ASIC1a is a voltage-insensitive cation channel, activated by H^+^ and abundantly expressed in CNS. It allows a Ca^2+^ influx in neurons and is involved in different hippocampal synaptic plasticity processes, including mGluRI-LTD [30,83]. ASIC1a pharmacological inhibition affects mGluRI-LTD magnitude in pyramidal neurons of hippocampal CA1 area [30].

Recent evidence supports that the functional interaction between mGluR1/5 and receptor tyrosine kinases (RTKs) can affect mGluRI-LTD expression [31]. ErbB tyrosine kinases (ErbB2, ErbB4)—receptors of the neurotrophic factors neuregulins (NRG)—finely control mGluR1 function [31,84], and shape mGluRI-LTD expression in pyramidal neurons of the hippocampal CA1 area and midbrain nigral dopamine neurons [85,86,87]. ErbB signaling bidirectionally manages mGluR1 expression, membrane docking, trafficking, proper effectors coupling, and function [84], and consequently NRG1-induced ErbB activation fosters mGluRI-LTD induction while, contrarily, ErbB inhibition reduces mGluRI-LTD magnitude either in the hippocampus or in midbrain nigral DA neurons [31,85,86,87]. Regarding other RTK roles in mGluRI-LTD modulation, it has been reported that mGluR5 cross-talks with the epidermal growth factor receptor (EGFR) and that EGFR inhibition decreases mGluR5 function [88,89,90], but precise EGFR-dependent roles in mGluRI-LTD remain to be ascertained.

Synaptic extracellular-matrix adhesion molecules could also contribute to shaping synaptic plasticity. While a large number of synaptic adhesion molecules have been identified, current knowledge of their involvement in mGluRI-LTD induction is rather limited. It has been reported that Ephrins and their receptors Ephrin-B2, by interacting with mGluR1 and mGluR5, cooperate in the induction of mGluRI-LTD at hippocampal CA3-CA1 synapses [91]. N-cadherines and their partners catenins also partake in the induction phase of hippocampal mGluRI-LTD, via a cofilin-mediated actin reorganization affecting AMPAR trafficking [92]. Moreover, a recent study has unveiled neuroligin 1 (NLG1) contribution to mGluRI-LTD, describing enhanced mGluRI-LTD magnitude at hippocampal CA3-CA1 synapses in NLG1^+/−^ mice, probably due to decreased GluA2 tyrosine de-phosphorylation [93]. 

**Figure 3:**
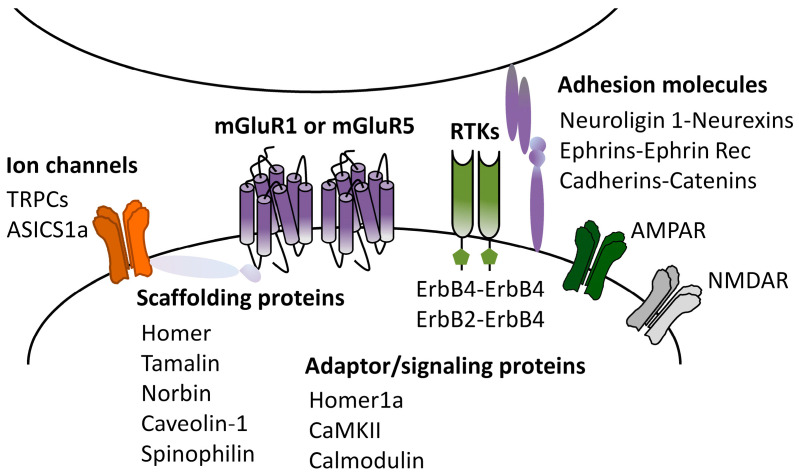
mGluRI partners affecting mGluRI-LTD. Scheme of mGluR1- and mGluR5-interacting proteins (physical and functional interactors) with modulatory roles on mGluRI-LTD.

Overall, cumulative data highlight that mGluR1- and mGluR5 partners can critically affect mGluRI-LTD induction (Figure 3). The list of such physical and functional mGluRI interactors will feasibly be updated in the future. Along with the recognition of the functional roles of each protein–protein interaction in general mGluRI function, it would be essential to unveil their unappreciated roles in mGluRI-LTD modulation. This would provide a better definition of the complex picture of the molecular mechanism underlying mGluRI-LTD, with potential significant implications in understanding the physiological and pathological mGluRI-dependent synaptic plasticity.

### 2.3. mGluRI-LTD Types and Their Functional Significance

Along with the investigation on the molecular mechanisms underlying different mGluRI-LTD forms, other studies pointed at deciphering the functional significance of mGluRI-LTD in various brain areas, providing partial comprehension of mGluRI-LTD roles in physiological brain functions.

*mGluRI-LTD in the cerebellum*—mGluRI-LTD has been firstly reported in the cerebellum at PF-PC synapses as an LTD induced by the activation of a glutamatergic metabotropic “quisqualate-sensitive receptor”, then proved to correspond to mGluR1 [94,95,96,97]. The mGluR1 role in cerebellar mGluRI-LTD has been then validated in mGluR1 KO mice and with broad-spectrum mGluRs antagonists or mGluR1 antibodies [96,97,98]. Cerebellar mGluRI-LTD relies on AMPARs endocytosis, induced by PKC-dependent GluR2 subunit phosphorylation, that weakens the AMPARs bond to scaffolding proteins [99,100,101]. Another mechanism, mainly involved in the initial induction phase, consists of the mGluRI-induced synthesis of eCBs, which reduce glutamate release by presynaptic CB1 [102,103,104]. 

Earlier evidence supports that mGluRI-LTD at cerebellar PF-PC synapses underlies motor learning. mGluR1 genetic deletion causes ataxia, deficit in motor coordination and eye-blink conditioning (a cerebellum-related learning process), in parallel with mGluRI-LTD impairment [96,97,104]. Such synaptic and motor learning deficits are both rescued by conditional mGluR1 re-expression only in the Purkinje cell [105]. Other recent evidence strengthens cerebellar mGluRI-LTD role in motor learning, such as eye-blink conditioning [106] and rotarod task performances [42].

*mGluRI-LTD in the hippocampus*—mGluRI-LTD has been described at all levels of the tripartite hippocampal circuitry, connecting the perforant pathway (PP), dentate gyrus (DG), cornus ammonis 3 (CA3), and cornus ammonis 1 (CA1) areas, i.e., PP-DG, DG-CA3, and CA3-CA1 synapses. mGluRI-LTD at CA3-CA1 synapses is the most investigated mGluRI-LTD type. It requires both mGluR1 and mGluR5 activation, and relies on AMPARs endocytosis [18,40]. Intracellular mechanisms, downstream of mGluRI activation, encompass various kinases pathways, including ERK1/2, PI3K-Akt-mTOR, and MAPKs, which foster the *de novo* synthesis of proteins instrumental to LTD expression/maintenance (named “LTD proteins”), including the immediate early gene product activity-regulated cytoskeleton-associated (Arc), microtubule-associated protein 1B (MAP1B) or striatal-enriched protein tyrosine phosphatase (STEP) [44,107,108]. Finally, AMPAR endocytosis relies on mGluRI-dependent STEP-induced GluA2 dephosphorylation on Tyr residues. While the *de novo* protein synthesis is instrumental to mGluRI-LTD in young adult rodents, at neonatal stages, mGluRI-LTD appears to be protein synthesis-independent and mainly reliant on presynaptic mechanisms [109], thus indicating a developmental switch in synaptic mechanisms underlying mGluRI-LTD at hippocampal CA3-CA1 synapses [108,109]. mGluR1/5 stimulation at hippocampal CA3-CA1 synapses also causes LTD of NMDAR-mediated transmission, by fostering NMDARs lateralization in PSD [50,51]. mGluRI-LTD in the DG at medial PP to granule cells (GC) synapses (PP-GC) can be induced by mGluRI agonists or synaptic mGluRI activation, via partially overlapping mechanisms [110,111,112], since mGluR1 and mGluR5 cooperation is required for chemical LTD, while mGluR1 mainly prompts LFS-induced LTD [112]. mGluRI-activated signaling pathways include PKC and MAPK [113,114], and result from Ca^2+^ increase in the post-synaptic compartment, possibly via L-type Ca^2+^ channels.

mGluRI stimulation induces LTD of the NMDARs-mediated transmission at hippocampal DG-CA3 synapses, which is reliant on a mGluR5-mediated exchange of GluN2B to GluN2A subunits [115].

mGluRI-LTD at hippocampal CA3-CA1 synapses is considered the biological substrate underlying novelty detection and object-place learning. It is endogenously induced in behaving rodents during the exploration of environments containing novel objects, and object recognition is impaired by manipulations preventing mGluRI-LTD expression in CA1 pyramidal neurons [85,116,117,118,119,120,121]. Additionally, hippocampal mGluRI-LTD at CA3-CA1 synapses contributes to cognitive flexibility, consisting of the acquisition of new memories and adjustments of behaviors based on updated spatial information [122,123,124,125]. Transgenic mice with abnormal mGluRI-LTD (either low or high) display deficits in reversal spatial learning [124,125,126,127], thus proper mGluRI-LTD magnitude is required for normal cognitive flexibility. Hippocampal LTD at PP-DG synapses has been proposed to underlie learning and memory of spatial information [128,129,130].

*mGluRI-LTD in the dorsal striatum (dST)*—mGluRI-LTD of corticostriatal glutamatergic transmission in medium spiny neurons (MSNs) of dST is selectively reliant on mGluR1 activation in adult rodents [41,131], while mGluR5 can also interplay in younger animals [132]. Striatal mGluRI-LTD requires the parallel activation of mGluRI and dopaminergic D2 receptor, by DA released from nigrostriatal terminals, that globally increase intracellular Ca^2+^ levels in MSNs, either through intracellular mobilization or via L-type Ca^2+^ channels-mediated influx, then fostering eCBs production and the CB1-induced reduction of glutamate release [54,133,134,135]. mGluRI signaling in striatal astrocytes can also cooperate to corticostriatal LTD forms in MSNs. Recently, it has been reported that astrocyte mGluR5 signaling contributes to shape the HFS-induced glutamatergic LTD in MSNs of a direct pathway, by promoting ATP release from astrocytes and adenosine A1 activation on MSNs [65].

The dST is involved in essential learning and memory processes, including motor learning, cognitive flexibility, and instrumental conditioning underlying goal-directed behaviors and habit formation [136,137,138]. Along with other synaptic plasticity forms, corticostriatal mGluRI-LTD can contribute to such learning and memory processes [136,139]. The link between corticostriatal mGluRI-LTD and motor learning is also sustained by evidence in Parkinson’s disease (PD) animal models showing corticostriatal mGluRI-LTD impairment in association with motor deficits, that are ameliorated by promoting mGluRI-LTD expression [41,131,134].

*mGluRI-LTD in the nucleus accumbens (NAc)*—mGluRI-LTD of AMPAR-mediated transmission is also observed in MSNs in the nucleus accumbens (NAc). The induction mechanisms and loci of induction appear distinct if mGluRI-LTD is triggered by mGluR1 or mGluR5. mGluR1-induced LTD relies on changes in AMPARs subunits composition, and the exchange of native Ca^2+^-permeable AMPARs (CP-AMPARs) with lower conductance Ca^2+^-impermeable AMPARs (CI-AMPARs) [53]. This mGluR1-LTD form is especially observed in potentiated synapses, as following exposure to psychostimulants or drugs of abuse [53,140,141]. mGluR5-induced LTD is mediated by the eCB-induced reduction of glutamate release [142,143,144]. Along with other synaptic plasticity forms in the brain reward system, mGluRI-LTD in NAc MSNs contributes to reinforcement learning for natural rewards, such as drinking, eating, and mating, or for addictive drugs, that result in reward-seeking behaviors [145,146].

*mGluRI-LTD in ventral tegmental area (VTA)*—mGluR1 activation in dopamine (DA) neurons of the ventral tegmental area (VTA) depresses glutamatergic synaptic transmission, producing in naive synapses a transient depression or LTD, also based on investigated rodent species [52,147]. Reliable mGluRI-LTD in VTA DA neurons of mice is reported in psychostimulants-potentiated synapses, and is mediated by the mGluR1-induced GluA2 synthesis and incorporation in surface-exposed AMPARs that become Ca^2+^-impermeable and less conductive [148]. mGluRI-LTD in VTA DA neurons cooperates with accumbal mGluRI-LTD to direct goal-oriented behaviors and reinforcement to rewards, with major relevance in the context of drug addiction [44,146].

*mGluRI-LTD in substantia nigra pars compacta (SNpc)*—mGluRI-LTD of AMPAR-mediated transmission has been reported in SNpc dopamine neurons [86,87]. Nigral mGluRI-LTD is reliant on mGluR1 stimulation, and overt in various rodent species (Wistar rats and C57BL6 mice) [86]. While precise underlying mechanisms are still unclarified, it has been proven that nigral mGluRI-LTD requires endogenous ErbB tyrosine kinases activity [86,87]. The physiological relevance of nigral mGluRI-LTD is currently unknown. The nigrostriatal DA pathway contributes to goal-oriented behaviors and cognitive functions, including reward/aversion-based learning, mental flexibility, and habit formation [149,150,151,152,153,154]; thus, future research might state if mGluRI-LTD in SNpc DA neurons contributes to such brain processes.

*mGluRI-LTD in lateral habenula (LHb)*—mGluRI-LTD of AMPAR-mediated transmission has also been described in LHb neurons, due to the mGluR1-induced eCB-mediated reduction of glutamate release, subsequent to PKC and presynaptic CB1 stimulation [155]. Habenular mGluRI-LTD has been proposed as a synaptic mechanism contributing to tune LHb neurons’ firing activity, with implications in LHb-dependent encoding of motivated states, i.e., reward or aversion [155]. 

*mGluRI-LTD in cortex*—mGluR1 stimulation mediates LFS-induced LTD in the anterior cingulate cortex (ACC) [156]. Studies in animals and humans demonstrate that ACC concurs, with other cortical areas, to pain perception and chronic pain conditions [157]. It has been proposed that mGluR1-LTD partakes in pain states, as it is impaired following the peripheral amputation of the distal tail in mice, and that mGluR1 activation may ameliorate pain or associated brain dysfunctions [156].

The thalamo-cortical synapses in the auditory cortex (AC) show mGluRI-LTD reliant on the selective mGluR5 activation [158]. Furthermore, in the perirhinal cortex, mGluRI cooperate with mGluRII and NMDARs in LTD induction in layer II/III neurons [159]. Such LTD form, due to the functional interaction between different classes of synaptically activated Glu receptors, could be involved in recognition memory [160]. Different mGluRI-LTD types are summarized in Table 1.

## 3. mGluRI-Dependent LTD in Pathology

Unbalanced mGluRI-dependent LTD represents a shared synaptic signature of different neurological and psychiatric disorders. In this section, we will summarize current evidence on mGluRI-LTD deviances in animal models of neurodevelopmental disorders associated with autism spectrum disorders, genetic intellectual disabilities, or schizophrenia, as well as mGluRI-LTD alterations in major depressive disorder, addiction, or neurodegenerative diseases, such as Alzheimer’s disease and Parkinson’s disease.

### 3.1. Autism Spectrum Disorders, Genetic Intellectual Disabilities, and Schizophrenia 

Autism spectrum disorders (ASDs) are a heterogeneous group of neurodevelopmental disorders, the core features of which are persistent deficits in social interaction and communication, restricted patterns of behavior and interests, as well as repetitive/stereotyped activities. ASDs encompass idiopathic or syndromic cases, with the latter caused by monogenic alterations, such as fragile X syndrome (FXS), tuberous sclerosis complex (TSC), Angelman’s syndrome (AS), and Rett’s syndrome (RS). Autistic traits and intellectual disabilities are also associated with other diseases with polygenic or more complex etiological mechanisms. In spite of heterogeneous genetic alterations, ASDs symptomatology is assumed to hang on converging mechanisms which regulate synapse formation/elimination, synaptic transmission, and plasticity. Actually, as described below, mGluRI-LTD alterations represent a commonality in different animal models of ASD and intellectual disability.

Fragile X syndrome (FXS)—the most common inherited form of intellectual disability and autism—is caused by transcriptional silencing of the fragile X mental retardation 1 (FMR1) gene, coding for the fragile X syndrome retardation protein (FMRP) [160,161]. mGluRI dysregulation, typically associated with abnormal mGluR5 activity, has been consistently reported in FXS animal models, thus inspiring the “mGluR theory” that accounts for synaptic, behavioral, and cognitive alterations related to this disorder, and for rescue effects caused by mGluR5 normalization [162,163,164,165].

Increased mGluRI-LTD at hippocampal CA3-CA1 synapses is a core synaptic feature of FXS, reliably stated in several FXS rodent models and clearly associated with FXS-related cognitive dysfunctions [166,167,168,169,170]. Decades of intense research have partially unveiled molecular mechanisms underlying aberrant hippocampal mGluRI-LTD in FXS models [161]. The most striking outcome concerns a pathological conversion of mGluRI-LTD induction mechanisms, as in physiological conditions in mature synapses, it requires the *de novo* synthesis of proteins instrumental to LTD induction (LTD proteins), whereas it becomes protein synthesis-independent in FXS rodent models (feasibly because expression levels of such LTD proteins are already high) [161,166,167,171]. Another key pathological feature reported in the hippocampus of FXS models is altered mGluR5 coupling to signaling proteins, due to a compromised bond to scaffolding Homer long proteins (1b/c, 2, and 3) and increased Homer1a link. Such mGluR5-Homer uncoupling is linked to aberrant constitutive mGluRI activity and protein synthesis-independence of the exacerbated mGluRI-LTD at hippocampal CA3-CA1 synapses [172]. Abnormal activation of the PI3K/AKT/mTOR pathway has been associated with different FXS phenotypes, including the exaggerated mGluRI-LTD magnitude at hippocampal CA3-CA1 synapses [173]. Beyond the hippocampus, increased mGluRI-LTD is also overt in the cerebellum at PF-PC synapses of FXS animal models [174,175]. Such synaptic defect is assumed as the pathological substrate of deficits in cerebellar learning processes, such as the conditioned eye blink, that is similarly impaired in *Fmr1* KO mice and FXS patients [175]. 

Tuberous sclerosis complex (TSC) is a multisystem genetic disorder caused by mutations in the *Tsc1* gene (9q34) or the *Tsc2* gene (16p13.3) encoding the tumor suppressor proteins, hamartin, and tuberin, respectively [176,177]. TSC typical features are benign tumors diffused in several organs (brain, eyes, heart, kidney, lung, liver, and skin), and in most cases neuropsychiatric symptoms, i.e., cognitive disabilities, behavioral alterations, autism, and epilepsy [178]. Hamartin and tuberin form a heterodimer complex—TSC1/2 complex—that physiologically represses protein translation, by directly inhibiting mTOR. Thus, dysfunctional TSC1/TSC2 complex prompts abnormal protein translation, cell growth, and proliferation [179,180]. Some studies have analyzed the impact of *Tsc1* or *Tsc2* deletion on hippocampal mGluRI-LTD, giving insights on the physiological Tsc1/Tsc2 role in synaptic plasticity and on synaptic defeats underlying TSC pathology. Genetic *Tsc2* ablation in mice impairs mGluRI-LTD at hippocampal CA3-CA1 synapses [181], and such synaptic dysfunction can be rescued by boosting mGluR5 activity via an mGluR5 positive allosteric modulator (PAM), that also ameliorates cognitive and behavioral deficits [181]. Moreover, acute local *Tsc1* gene silencing in mice damages hippocampal mGluRI-LTD in CA1 area pyramidal neurons [182], and, accordingly, hippocampal mGluRI-LTD induction is prohibited by *Tsc1* or *Tsc2* conditional deletion in pyramidal neurons [183]. Overall, current evidence demonstrates that *Tsc1* or *Tsc2* mutations similarly abolish mGluRI-LTD at hippocampal CA3-CA1 synapses, without affecting other synaptic plasticity forms, such as HFS-induced LTP or NMDAR-dependent LTD. This further corroborates the notion that a selective mGluRI-LTD dysfunction, rather than general synaptic deficits, is a commonality of ASD mechanisms. 

Angelman’s syndrome (AS) is a neurodevelopmental disorder caused by mutations or deletions of the maternally inherited *Ube3a* gene and characterized by typical behavioral alterations, such as persistent social smiling, giggling, and mouthing behaviors, associated with speech impairments, mental retardation, epilepsy, abnormal EEGs, atypical sleep patterns, and hyperactivity. Such neurological deficits feasibly arise from synaptic dysfunctions, in light of the key role of Ube3a in normal synaptic development and plasticity [184,185]. The investigation of mGluRI-LTD dysregulation in AS models is confined to a study describing increased mGluR5-LTD at hippocampal CA3-CA1 synapses in slices of the maternal *Ube3A*-deficient AS mouse model [186]. Abnormal mGluRI-LTD magnitude could result from increased mGluR5 signaling efficiency, as mGlu5 coupling to Homer 1b/c proteins is enhanced in the hippocampus of this AS mouse model [186]. 

Rett’s syndrome (RTT) is a neurodevelopment disorder caused by *de novo* mutations in the methyl CpG binding protein 2 (Mecp2) gene, implied in gene transcription regulation, synapse development, and synaptic plasticity [187]. RTT is characterized by severe global regression in infant girls, resulting in lifelong severe mental retardation, language deficits, purposeful hand use, and, often, epilepsy and autism. Some studies have identified developmental-related dysregulations of hippocampal mGluRI-LTD in an RTT animal model, the *Mecp2* KO mice. Specifically, mGluRI-LTD magnitude at hippocampal CA3-CA1 synapses appears preserved in adolescent (~P30) *Mecp2* KO mice, despite a modification in underlying molecular mechanisms, with mGluRI-LTD becoming protein synthesis-independent [188,189]. Otherwise, hippocampal mGluRI-LTD alteration is overt in young adulthood (~P60) RTT mice, in relation to a reduced mGluR5 expression in the hippocampus at this age [190]. 

Phelan–McDermid syndrome (PMS) (or 22q13.3 deletion syndrome) is a rare neurodevelopmental disorder characterized by generalized developmental delay, intellectual disability, absent or delayed speech, autism, seizures, neonatal hypotonia, physical dysmorphic features, and recurrent medical comorbidities [191,192]. A more frequent cause is the deletion of the chromosomal region 22q13.3 including the Shank3 gene; supporting Shank3-related structural and functional alterations in the glutamatergic synapses might underlie the neurological and behavioral deficits observed in patients [191,192]. Investigations in animal models harboring Shank3 deletion or mutations provided insights into Shank3-induced regulation of mGluRI functions, including mGluRI-LTD. The main findings support that ASD-associated Shank3 mutations impair mGluRI-LTD induction at hippocampal CA3-CA1 synapses. Actually, hippocampal mGluRI-LTD is prevented in brain slices of mice with Shank3 exon 21 mutations [193] or with ASD-associated Shank3 R87C and R375C point mutations [194]. Differently, mGluRI-LTD magnitude at hippocampal CA3-CA1 synapses is less affected by Shank3 exon 21 deletion [195]. Shank3 deletion also impairs mGluR5 levels in PSD and HFS-induced LTD at corticostriatal synapses in dST MSNs [196]. Of note, mGluR5 stimulation with a PAM rescues the impaired HFS-LTD and ameliorates ASD-relevant behaviors [196].

The 16p.11 microdeletion syndrome—Besides monogenic alterations leading to well-recognized syndromes with a high autism prevalence (especially FXS and TS), chromosomal copy number variations (CNVs) have been found in 5–10% of ASD patients. Variation at human chromosome 16p11.2, the most common of these CNVs, accounts for 0.5–1% of all ASD cases [197], and is associated with language impairment, intellectual disability, autistic traits, anxiety, and epilepsy [198]. A recent study described subtle alterations in hippocampal mGluRI-LTD in a mouse model of human chr16p11.2 microdeletion, the 16p11.2^+/−^ mouse [199]. Specifically, while mGluRI-LTD magnitude at hippocampal CA3-CA1 synapses remains unchanged, it becomes protein synthesis-independent, supporting that, in spite of similar downscaling, alterations in molecular mechanisms underlying hippocampal mGluRI-LTD represent the main synaptic defect associated with 16p.11 microdeletion [199].

Down’s syndrome (DS), caused by chromosome 21 trisomy, is a common cause of intellectual disability, with overt learning and memory deficits, also associated with hippocampal dysfunctions. A recent study in a DS animal model, the Ts1Cje mouse, demonstrates enhanced mGluRI-LTD magnitude at hippocampal CA3-CA1 synapses in mouse brain slices [200]. 

Schizophrenia—Latest evidence suggests that shared synaptic alterations might underlie ASD and schizophrenia, a severe disease with a complex symptomatology characterized by positive (locomotor hyperactivity, aberrant sensory-motor functions), negative (social avoidance, apathy, depression), and cognitive deficits. To date, evidence on mGluRI-LTD dysregulation in schizophrenia models is quite limited. A study analyzing mGluRI function in a mouse line harboring a deletion of dysbindin 1 (dys-1), a risk gene for schizophrenia, has reported reduced mGluRI-LTD magnitude at hippocampal CA3-CA1 synapses [121]. In line with the hippocampal mGluRI-LTD contribution to cognitive processes, dys-1 genetic deletion produces deficits in object recognition memory and spatial learning (besides impaired mGluRI-LTD), that are reversed by a treatment with an mGluR5 PAM [121]. 

Overall, current knowledge identifies hippocampal mGluRI-LTD dysregulation as a shared synaptic signature of several neurodevelopmental disorders associated with autism, intellectual disabilities, and behavioral alterations. Strikingly, opposite pathological deviances (increased and decreased mGluRI-LTD magnitude) are visible in diverse disorders, in spite of convergent cognitive deficits and behavioral alterations. Exacerbated hippocampal mGluRI-LTD is overt in FXS, AS, and DS animal models, whereas reduced mGluRI-LTD has been found in TSC, PMS, RTT, and the schizophrenia-related sdy1 models; therefore, bidirectional shifts from an adequate mGluRI-LTD magnitude at hippocampal CA3-CA1 synapses equally converge in brain dysfunctions, ASD-relevant cognitive deficits, and behavioral alterations. In addition to better elucidate disease-specific mechanisms instrumental to hippocampal mGluRI-LTD dysregulation, detailed investigations are warranted to clarify if mGluRI-LTD abnormalities extend to other ASD-relevant brain areas, including striatum and midbrain dopamine nuclei, which are increasingly involved in ASD symptomatology but so far poorly investigated.

### 3.2. Alzheimer’s Disease 

Alzheimer’s disease (AD) is the most common cause of dementia and cognitive impairment in the aging population. Synaptic dysfunctions are early pathological AD features, preceding the formation of amyloid plaques and tau deposits, and neuronal loss [201]. Abnormal synaptic plasticity, mainly reported in the hippocampal area of AD animal models, is believed to underlie AD-related cognitive deficits and behavioral alterations. Evidence in AD models proves an enhanced mGluRI-LTD magnitude at hippocampal CA3-CA1 synapses. Pathogenic amyloid-β peptides (Aβ) can directly promote mGluR5-LTD of AMPAR-mediated transmission, by fostering AMPARs endocytosis [202,203,204,205,206,207]. Such Aβ-induced LTD occludes the subsequent mGluRI-LTD due to pharmacological mGluRI activation [202,203,208], suggesting shared mechanisms of LTD expression. In addition, Aβ can foster hippocampal mGluRI-LTD by favoring synaptic mGluR1/5 activation by glutamate, by blocking extracellular glutamate reuptake [209]. Aβ-mediated mGluRI-LTD facilitation at hippocampal CA3-CA1 synapses also involves ASIC1a channels, as demonstrated in AD mouse models [210]. Aβ also facilitates mGluRI-LTD induction in the dentate gyrus at medial PF-GC synapses [211]. Besides the Aβ impact on mGluRI-LTD, additional evidence from a transgenic AD animal model supports that mGluRI-LTD dysfunction is a pathological synaptic signature of AD. The mGluRI-LTD at hippocampal CA3-C1 synapses has been investigated in the transgenic Tg2576 AD mouse model, and there is indication of an either unchanged [211] or amplified [210] mGluRI-LTD magnitude. Moreover, in a different AD animal model, the APP/PS1 mouse, hippocampal mGluRI-LTD induction is prevented, as mGluRI stimulation only induces a transient synaptic depression of fEPSPs at CA3-CA1 synapses, in spite of a stable LTD [212]. Globally, the current scenario supports mGluRI-LTD dysregulation in AD pathology, despite data appearing not to be fully exhaustive. While reliable evidence proves the Aβ facilitator role in hippocampal mGluRI-LTD, findings from transgenic mice appear divergent, thus further research is warranted to find a better definition of mGluRI-LTD abnormalities in AD models. 

### 3.3. Stress-Induced Depressive Phenotypes 

Major depression, one of the most common and debilitating brain illnesses, has recently been linked to mGluRI dysregulation [213]. Depressed patients have reduced mGluR5 density in several brain areas, including cortex and hippocampus [214] (considered a compensatory modification) [213], while rodent models subjected to stress protocols inducing a depressive-like phenotype mainly display hippocampal mGluR1 and mGluR5 upregulation [215,216] or striatal mGluR5 increase [217]. Hippocampal mGluRI-LTD dysregulation is emerging as a stress-induced synaptic adaptation. Actually, acute restraining stress gates mGluR1-LTD induction in the hippocampal CA1 area [218], and mGluR5-LTD at hippocampal CA3-CA1 synapses are potentiated in susceptible mice with a depressive phenotype due to chronic social defeat stress (CSDS), a stress protocol caused by exposition to a strange aggressive mouse [219,220].

### 3.4. Addiction

Maladaptive synaptic plasticity in reward-associated brain areas (mainly VTA and NAc) is instrumental to the establishment of addiction-related behaviors, and the mGluRI-LTD of glutamatergic transmission partakes in such pathological synaptic adjustments [146,221]. As previously mentioned, mGluRI-LTD in NAc MSNs has distinct induction mechanisms if driven by mGluR1 or mGluR5. Such subtype-specific mGluRI-LTD forms are differently affected by addictive drug exposure and can diversely contribute to synaptic adaptations underlying reward-seeking behaviors or the incubation of craving for drug abuse [221]. 

mGluR5-LTD in MSNs—due to eCB-dependent reduction of glutamate release—is abolished in mice acutely exposed to drugs of abuse (cocaine and delta 9-tetrahydrocannabinol, THC) or during withdrawal from repeated drug exposures [143,144,222,223]. Cocaine-induced mGluR5-LTD impairment is due to reduced mGluR5 surface expression in MSNs [144]. mGluR5-LTD in NAc D1-expressing MSNs directly underlies the formation of addictive behaviors, as conditional mGluR5 deletion in D1-expressing MSNs, beyond LTD, prevents the appearance of rewards-seeking behaviors, either for drug (cocaine and ethanol) or natural (saccharin) rewards [224]. 

mGluR1-LTD, reliant on mGluR1-induced changes of AMPARs subunitscomposition in MSNs, is instead inducible in NAc MSNs of rodents acutely exposed to addictive drugs or that have undergone an incubation of craving [221,225]. Of note, mGluR1-LTD reduces the expression of incubated cravings for cocaine [53,140,141] and methamphetamine [226]. mGluR1-LTD can also counteract addictive drugs-induced synaptic plasticity in VTA DA neurons. Indeed, cocaine exposure drives a potentiation of excitatory transmission in VTA DA neurons [227] that is downscaled by mGluRI-LTD induction [52,147,148]. Overall, this supports that eliciting mGluR1-LTD, in NAc and VTA, by mGluR1 PAM could rebalance addictive drug-induced plasticity and reduce incubated cue-induced cocaine-seeking [53,221].

### 3.5. Parkinson’s Disease

Parkinson’s disease (PD) is an age-related neurodegenerative disease, characterized by severe motor deficits (i.e., rigidity, bradykinesia, resting tremor, and postural instability) and debilitating non-motor symptoms, including autonomic dysfunctions, cognitive abnormalities, psychiatric symptoms (depression and anxiety), and sleep disorders [228,229]. Modifications in mGluR1 and mGluR5 levels have been reported in different basal ganglia nuclei, the brain circuitry whose dysregulation, starting from SNpc dopamine neuron degeneration, is mainly liable for PD symptoms [230,231]. Corticostriatal mGluRI-LTD deficits are overt in PD animal models, in line with the evidence that striatal DA release is instrumental to proper LTD expression in MSNs [41,134,232]. Corticostriatal mGluRI-LTD loss observed in PD models is ameliorated by the DA precursor, L-DOPA, or D2 agonists plus inhibitors of eCB degradation (that boost eCB levels), and such pharmacological treatments in parallel recover motor deficits [134,232]. Besides dST, some evidence describes that the mGluRI-LTD at hippocampal CA3-CA1 synapses is also affected in PD animal models. Actually, the mGluRI-LTD induction at CA3-CA1 synapses is prevented in mouse hippocampal slices treated with the toxin MPP^+^ [233]. Deficits in hippocampal mGluRI-LTD might contribute, along with other synaptic deficits previously reported in PD animal models [234,235], to learning and memory deficits associated with non-motor PD symptoms. 

## 4. Conclusions and Perspectives

Extensive evidence identifies mGluRI-LTD as a key process by which mGluRI can broadly affect neuronal connectivity, directing complex brain functions and behaviors. Emerging data suggest that, along with conventional and well-known molecular mechanisms (iGluRs endocytosis, changes in iGluRs subunits’ composition, and eCB-regulated glutamate release), other processes could partake in mGluRI-LTD expression. Future research may uphold the relevance of such novel mechanisms, validating either astrocytes-dependent gliotransmitters release or the activation of intracellular mGluRI located in neuronal compartments as starting events contributing to mGluRI-LTD induction. 

The essential roles of mGluRI-interacting proteins (physical and functional partners) in proper mGluRI-LTD expression have been recently reported. The current picture outlines that mGluRI interplay with scaffolding/adaptor proteins, extracellular adhesion molecules, ion channels, and ErbB receptor tyrosine kinases definitively rule physiological mGluRI-LTD magnitude. 

Knowledge on physiological mGluRI-LTD has been advanced with an increasing list of brain areas expressing this form of synaptic plasticity, and although the exact definition of the functional relevance in each area warrants more investigation, to date it is accepted that mGluRI-LTD is the biological substrate of essential learning and memory processes, thus guiding cognitive processes and learning-based behaviors.

mGluRI-LTD dysregulation stands as a key synaptic signature in several brain disorders, and an optimal range of mGluRI-LTD magnitude is required to sustain proper brain functions. This is particularly overt for mGluRI-LTD at hippocampal CA3-CA1 synapses, whose deviance in either direction (enhancement or reduction) is associated with similar cognitive and behavioral deficits, as mostly observed in animal models of genetic intellectual disabilities and autism. Thus, mGluRI modulation in opposite directions to counterbalance mGluRI-LTD to homeostatic (physiological) levels could be feasibly considered a valuable therapeutical strategy for diverse neurological and psychiatric disorders. Actually, even though mGluR1 and mGluR5 can influence brain processes in multiple ways, mGluRI-LTD regulation by PAM or NAM (based on the pathological mGluRI-LTD shift) can restore synaptic homeostasis, rescuing cognitive and behavioral alterations. This is demonstrated by robust evidence, mainly from animal models of FXS and other neurodevelopmental disorders, that clearly proves that mGluRI modulators (so far targeting mGluR5) can correct hippocampal mGluRI-LTD deviations, simultaneously improving some cognitive and behavioral deficits [121,162,163,164,199].

Preclinical data have encouraged evaluations of mGluRI modulators in various human clinical trials for several brain diseases. While a general discussion on the clinical trials investigating mGluRI modulators in brain diseases is beyond the scope of this review, here it appears relevant to emphasize that outcomes from clinical trials in FXS, that is at the forefront of the clinical investigation of mGluRI modulators (namely mGluR5 NAMs), indicate that mGluR5 NAMs fail to ameliorate the most severe symptoms in humans [236], despite robust preclinical data in animal models. Nonetheless, such failures of FXS clinical trials, instead of questioning the factual pathological mGluRI relevance, should rather encourage a deeper investigation of molecular mechanisms underlying mGluRI dysregulation, to possibly disclose additional collateral processes that might limit the therapeutic efficacy of mere mGluR5 inhibition in humans, as well as reveal functional adaptations that might decrease the therapeutic efficacy during a chronic drug administration regimen.

In conclusion, by considering that mGluRI-LTD dysregulation is the foremost important synaptic feature in several brain illnesses, advances in the definition of the exact molecular mechanisms of induction and endogenous regulation that go awry in distinct diseases could help to establish novel and more effective therapeutical strategies. To address this point, research efforts should also be focused on novel mechanisms, including glia interplay, the compartmentalized mGluRI subcellular location, and the complex network of mGluRI interactors, and also look for potential brain area/synapse-specific distinct anomalies. Major awareness of mGluRI-LTD physiopathology would have great implications for a general understanding of brain functions in health and disease.

## Figures and Tables

**Table 1:** mGluRI-LTD.

Brain Area	Synapse/ Cellular Type	mGluRI Subtype	Main Mechanism of Induction	Physiological Functions	Former Refs	Dysregulation in Disease Models
Cerebellum	Purkinje cells	mGluR1	AMPARs endocytosis	Motor learning	[96]	
Hippocampus	CA3-CA1	mGluR1 mGluR5	AMPARs endocytosis	Novelty detection Object-place recognition learning Cognitive flexibility	[18,38,39,40,107]	Fragile X syndrome ↑ Tuberous sclerosis ↓ Angelman’s syndrome↑ Rett’s syndrome ↓ Down’s syndrome↑ Phelan–McDermid syndrome ↓ Schizophrenia ↓ Stress-induced depression ↑
	CA3-CA1	mGluR1 mGluR5	NMDA endocytosis	Spatial learning	[50,51]	
	PP-DG	mGluR1 mGluR5			[110,111,112]	
	DG-CA3	mGluR5	Changes in NMDARs composition		[115]	
Dorsal striatum	Corticostriatal/ MSNs	mGluR1 mGluR5 (D2R)	eCB-regulated Glu release	Motor-learning Goal-oriented behaviors	[41,54]	Parkinson’s disease ↓
Nucleus accumbens	MSNs	mGluR5	eCB-regulated Glu release	Rewards-seeking behaviors	[142]	Addiction (reward-seeking)
	MSNs	mGluR1	Changes in AMPARs composition	Rewards-seeking behaviors	[53]	Addiction (drug craving)
Ventral tegmental area	DA neurons	mGluR1	Changes in AMPARs composition		[52,147]	Addiction (reduces addictive drugs-induced plasticity)
Substantia nigra pars compacta	DA neurons	mGluR1			[86]	
Lateral habenula	LHb neurons	mGluR1	eCB-regulated Glu release		[155]	
Anterior cingulate cortex	Layer II/III Layer V Pyramidal neurons	mGluR1			[156]	Chronic pain conditions
Auditory cortex	Layer IV Pyramidal neurons	mGluR5			[158]	
Perirhinal cortex	Layer II/III Pyramidal neurons	mGluR1 mGluR5		Recognition memory	[159]	
